# Changes in Self-Reported Hunger Among Patients in Heroin-Assisted Treatment in Norway

**DOI:** 10.1192/j.eurpsy.2026.10491

**Published:** 2026-07-31

**Authors:** M. Leonhardt, Z. Gaulen

**Affiliations:** 1 Research Centre for Substance Use Disorders and Mental Illness, Inlandet Hospital Trust, Brumunddal; 2 Department of Addiction Medicine, Haukeland University Hospital, Bergen, Norway

## Abstract

**Introduction:**

Heroin-assisted treatment (HAT) is an evidence-based intervention for opioid use disorder (OUD) offered in select countries for patients unresponsive to standard treatments. It provides supervised medical heroin (diacetylmorphine) as an injection or in a tablet form in clinical settings. In 2022, Norway launched a five-year HAT model project, currently under comprehensive evaluation including medical, psychological, and social outcomes. Food security and hunger are emerging issues in addiction research but have scarcely been examined in the context of HAT.

**Objectives:**

This sub-study investigates changes in the self-reported frequency of hunger among HAT patients in Norway over the first 12 months of treatment, and examines whether food access, demographics, substance use, and clinic site predict variation in how often patients report feeling hungry.

**Methods:**

Self-reported data on hunger frequency and eating patterns were collected at baseline, 3, 6, and 12 months. Breakfast was provided at the Oslo clinic site but not in Bergen. Descriptive statistics characterized the cohort at baseline, and ordinal logistic regression was applied to examine changes in hunger frequency and the effects of predictors.

**Results:**

A total of 126 participants (Bergen n = 40; Oslo n=86) were recruited, predominantly male (80.2%) with a mean age of 45.5 years (SD 9.1). Most had primary or secondary education and stable housing. Baseline site differences were observed (Table 1).

**Table 1.** Characteristics of participants (N=126) at study sites Bergen and Oslo Table 1. Long description.

Lifetime Characteristic	Bergen (n = 40)	Oslo (n = 86)
Age, mean (SD), y	42.8 (7.8)	46.4 (10.2)
Male, No. (%)	28 (70.0)	73 (84.9)
Stable housing	33 (82.5)	63 (73.3)
Rare food access, No. (%)	8 (20.0)	26 (30.2)
Often food access, No. (%)	12 (30.0)	30 (34.9)
Always food access, No (%)	13 (32.5)	18 (20.9)
1 meal/day, No. (%)	8 (20.0)	20 (23.3)
2 meals/day, No. (%)	6 (15.0)	26 (30.2)
3 meals/day, No. (%)	11 (27.5)	24 (27.9)

Preliminary analyses showed that food access significantly predicted hunger frequency (coefficient = -0.67, p = 0.001), indicating that better food access was associated with reporting less frequent hunger. Analysis suggested further that other substance use than diacetylmorphine (coefficient = 0.75, p = 0.01) predicted lower hunger frequency. Breakfast consumption showed a significant effect (coefficient = 0.54, p = 0.02), implying that regular breakfast intake was linked to reduced hunger. Gender, age and time in treatment were not significant predictors.

**Conclusions:**

Findings suggest that better food access and regular breakfast consumption are associated with reduced frequency of hunger among patients in heroin-assisted treatment. Interventions to improve food security and meal routines may help to alleviate hunger in this population and contribute to improved quality of life during treatment.

**Disclosure of Interest:**

None Declared

## Long descriptions

### Table 1. Long description

The table consists of three columns: Lifetime Characteristic, Bergen (n = 40), and Oslo (n = 86).

* Age, mean (S D), y: Bergen 42.8 (7.8); Oslo 46.4 (10.2).

* Male, No. (%): Bergen 28 (70.0); Oslo 73 (84.9).

* Stable housing: Bergen 33 (82.5); Oslo 63 (73.3).

* Rare food access, No. (%): Bergen 8 (20.0); Oslo 26 (30.2).

* Often food access, No. (%): Bergen 12 (30.0); Oslo 30 (34.9).

* Always food access, No (%): Bergen 13 (32.5); Oslo 18 (20.9).

* 1 meal/day, No. (%): Bergen 8 (20.0); Oslo 20 (23.3).

* 2 meals/day, No. (%): Bergen 6 (15.0); Oslo 26 (30.2).

* 3 meals/day, No. (%): Bergen 11 (27.5); Oslo 24 (27.9).

Navigate back to Table 1..

